# A loss-of-function *AGTR1* variant in a critically-ill infant with renal tubular dysgenesis: case presentation and literature review

**DOI:** 10.1186/s12882-024-03569-z

**Published:** 2024-04-22

**Authors:** Aljazi Al-Maraghi, Waleed Aamer, Mubarak Ziab, Elbay Aliyev, Najwa Elbashir, Sura Hussein, Sasirekha Palaniswamy, Dhullipala Anand, Donald R. Love, Adrian Charles, Ammira A.S.Akil, Khalid A. Fakhro

**Affiliations:** 1 Laboratory of Genomic Medicine, Sidra Medicine, P.O. Box 26999, Doha, Qatar; 2 Department of Human Genetics-Precision Medicine in Diabetes Prevention, Sidra Medicine, P.O. Box 26999, Doha, Qatar; 3 Neonatology Division, Sidra Medicine, P.O. Box 26999, Doha, Qatar; 4 Genetic Pathology, Sidra Medicine, P.O. Box 26999, Doha, Qatar; 5 Anatomical Pathology, Sidra Medicine, P.O. Box 26999, Doha, Qatar; 6 College of Health and Life Sciences, Hamad Bin Khalifa University, P.O. Box 34110, Doha, Qatar; 7 Department of Genetic Medicine, Weill Cornell Medical College, P.O. Box 24144, Doha, Qatar

**Keywords:** Renal tubular dysgenesis, AGTR1, Middle East, Rare Mendelian disease, Whole genome sequencing

## Abstract

**Background:**

Renal tubular dysgenesis (RTD) is a severe disorder with poor prognosis significantly impacting the proximal tubules of the kidney while maintaining an anatomically normal gross structure. The genetic origin of RTD, involving variants in the *ACE, REN, AGT,* and *AGTR1* genes*,* affects various enzymes or receptors within the Renin angiotensin system (RAS). This condition manifests prenatally with oligohydramninos and postnatally with persistent anuria, severe refractory hypotension, and defects in skull ossification.

**Case presentation:**

In this report, we describe a case of a female patient who, despite receiving multi vasopressor treatment, experienced persistent hypotension, ultimately resulting in early death at five days of age. While there was a history of parental consanguinity, no reported family history of renal disease existed. Blood samples from the parents and the remaining DNA sample of the patient underwent Whole Genome Sequencing (WGS). The genetic analysis revealed a rare homozygous loss of function variant (NM_000685.5; c.415C > T; p.Arg139*) in the Angiotensin II Receptor Type 1 (*AGTR1*) gene.

**Conclusion:**

This case highlights the consequence of loss-of-function variants in *AGTR1* gene leading to RTD, which is characterized by high mortality rate at birth or during the neonatal period. Furthermore, we provide a comprehensive review of previously reported variants in the *AGTR1* gene, which is the least encountered genetic cause of RTD, along with their associated clinical features.

**Supplementary Information:**

The online version contains supplementary material available at 10.1186/s12882-024-03569-z.

## Background

Renal tubular dysgenesis (RTD) (MIM# 267,430) is a rare autosomal recessive disorder of renal tubular development that was first characterized in two stillborn siblings in 1983 [1]. The disease carries a poor prognosis and a high mortality rate due to the severity of the disease where patients may die in utero or soon after birth, despite the availability of high-quality clinical care. Although the exact prevalence of RTD is unknown, there are multiple reports of RTD cases [2, 3].

The underlying pathophysiology of RTD involves reduced intrauterine renal perfusion leading to dysgenesis of proximal tubule formation in the kidneys, with preservation of grossly normal kidney structure [4].The clinical manifestations of RTD include persistent fetal anuria with subsequent oligohydramnios in pregnancy, pulmonary hypoplasia, and skull ossification defects of the bone due to persistent hypotension [5]. In addition, typical pathological changes seen on kidney sections taken from affected patients show the incomplete development of renal proximal tubules. These changes are attributed to the consequences of hypoperfusion and renal ischemia in the absence of Angiotensin II (ANG II) production or function, a defect responsible for the severe refractory hypotension observed at birth [6].

Previous studies have demonstrated the fundamental role of Renin Angiotensin System (RAS) during fetal development of the kidneys. Physiologically, the RAS pathway regulates extracellular fluid volume and maintains blood pressure levels in the body [7]. Several variants in four different genes encoding RAS signaling proteins (*AGT*, *REN*, *ACE*, and *AGTR1*) have been described to cause RTD [8]. Variants in the *AGTR1* gene constitute approximately 8% of the reported mutations causing RTD [9].

The Angiotensin II Receptor Type 1 (*AGTR1*) gene encodes a receptor protein of the ligand angiotensin II, which is a potent vasopressor hormone in the RAS pathway [10]. The binding of ANG II to the Angiotensin II Type 1 receptor (AT1 receptor) promotes its activation, leading to vasoconstriction, sympathetic activity and aldosterone release from adrenals, ultimately increasing blood pressure [11]. Angiotensin II also regulates renal growth during fetal development [12].

Herein, we report a rare nonsense variant in the *AGTR1* gene detected through whole genome sequencing (WGS) in a neonate exhibiting persistent anuria and resistant refractory pulmonary hypoplasia, ultimately resulting in early lethality.

## Case presentation

The female patient, born to consanguineous parents (first degree cousins) with a family history of Oculocutaneous Albinism in the mother. This was the mother’s first pregnancy and antental ultrasound scans revealed oligohydramnios and Intra-Uterine Growth Retardation (IUGR). The patient was born prematurely at 36 weeks through an emergency cesarean section due to reduced fetal movement and failed induction. The baby was born weighing 2.0 kg with meconium stained liquor and Apgar scores were 6 and 9 at one and five minutes, respectively. The baby required minimal resuscitation and she was managed on continuous positive airway pressure (CPAP) in the first hour of life; however, within a few hours she deteriorated with bilateral pneumothoraces requiring chest drains, intubation, and ventilation. The patient was started on inhaled nitric oxide for hypoxic respiratory failure, and inotropes due to low blood pressure including dopamine, dobutamine, and epinephrine. The patient remained hypotensive with a mean blood pressure of 15–20 mmHg, which required the addition of hydrocortisone followed by vasopressin to improve her blood pressure. Her oxygen saturation measurements were 35%—45% in 100% FiO_2_. Supportive measures, including sedation, antibiotics, and fluids were administered. The patient didn’t have any urine output and she developed persistent hypoxia and hypotension, necessitating veno-arterial Extra Corporeal Membrane Oxygenation (ECMO) support on the second day of life, which led to an improvement in her oxygen saturation. However, the patients blood pressure remained low despite the ECMO and continuous inotropic support. While on ECMO, renal replacement therapy (CRRT) was initiated, effectively normalzing the creatinine levels, however the CRRT was discontinued due to the development of hypotension, resulting in progressive edema and fluid overload. Subsequently, the decision was made to decannulate and remove the ECMO support due to a substantial right-sided parenchymal hemorrhage and extra-axial hemorrhage observed on head ultrasound. The patient experienced coagulopathy, manifesting as oozing from the skin and chest tubes requiring multiple Fresh Frozen Plasma (FFP), cryoprecipitate, and red cell transfusions due to low hemoglobin, persistent thrombocytopenia and coagulopathy. On the fourth day, a multi-disciplinary team meeting, with the patient’s parents present, concluded to transition the patient from intensive care to comfort care with no further resuscitation. The patient was extubated the following day and passed away a few hours later.

Imaging studies that were done on the baby included: (1) Echocardiography, which showed a structurally normal heart but was associated with severe persistent pulmonary hypertension of newborn (PPHN) and complete right to left shunting across the ductus arteriosus; (2) Abdominal ultrasound, which showed non-specific bilateral echogenic kidneys; (3) Head ultrasound, which showed large left intra-parenchymal and extra-axial acute bleeding associated with mass effect.

**Fig. 1:**
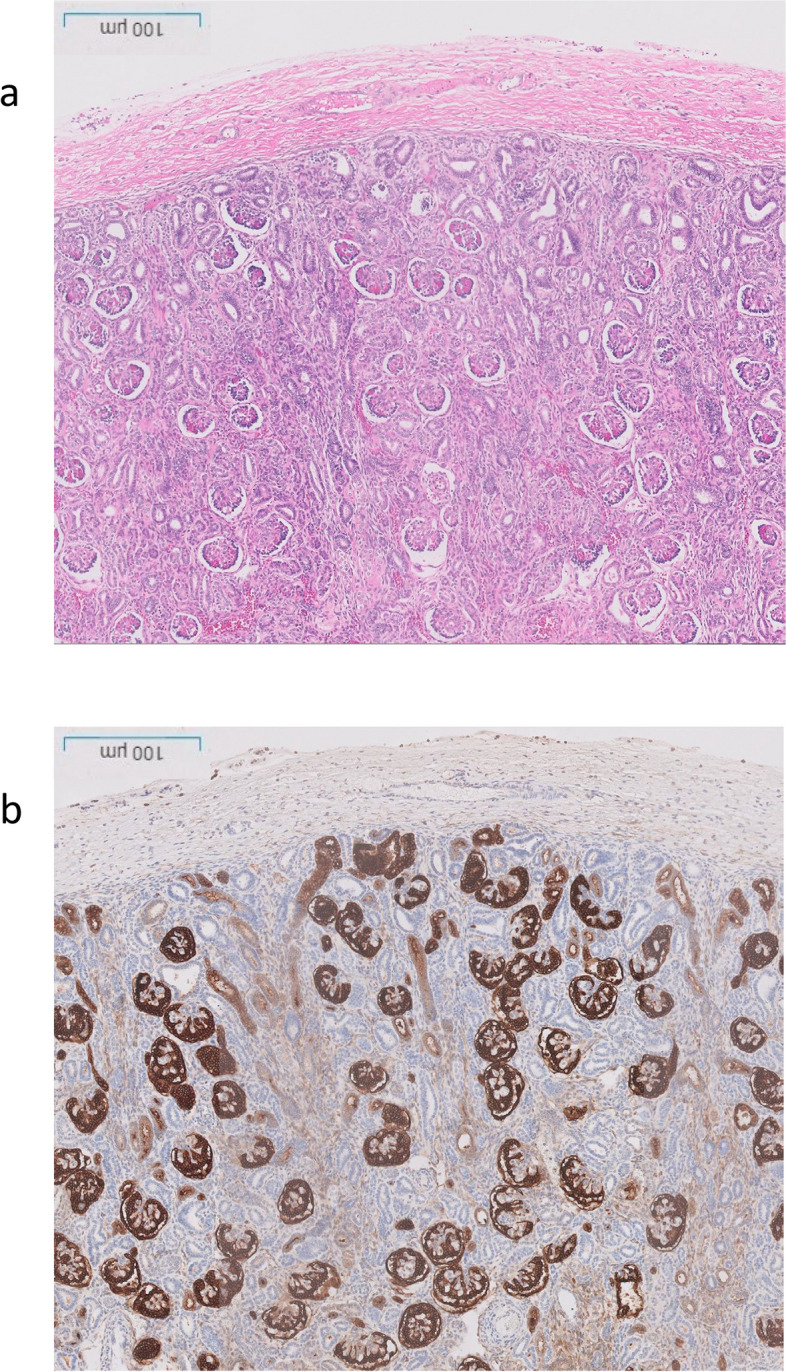
Renal histological characteristics. **a** H&E renal cortex, showing crowding of the glomeruli, with intervening tubules mainly of distal tubule type, and lack of proximal tubules. **b** CD10 highlighting the glomeruli and the Bowmans capsule, but normal proximal tubules are not seen, only weak staining of the ureteric buds

**Fig. 2:**
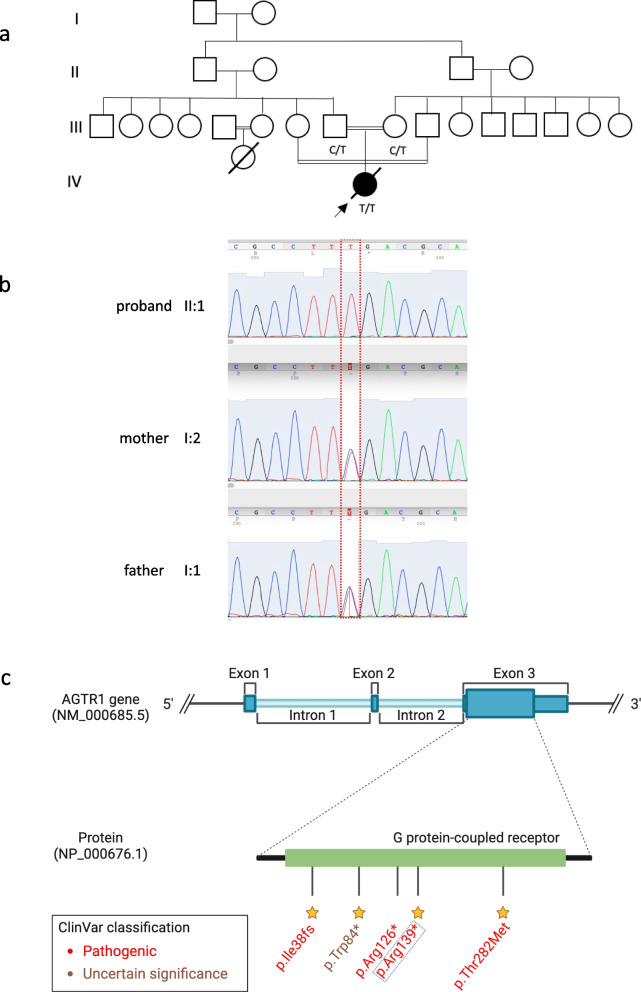
Patient characteristics and genetic findings. **a** Family pedigree of the patient along with genotypes of the nonesense *AGTR1* variant (c.415C > T; p.Arg139*). **b** Chromatogram of Sanger sequencing showing the variant position and genotypes of the 3 family members. **c** Schematic of *AGTR1* gene body with highlights of protein domains and reported ClinVar variants. The yellow stars refer to the staring system of ClinVar which indicate the review status of the variant

The post-mortem examination revealed mildly hypoplastic kidneys, moderate pulmonary hypoplasia, solid and poorly aerated lungs with diffuse alveolar damage, significantly reduced skull vault mineralization and bony development, indicative features of oligohydramnios sequence. Limbs exhibited some flexion changes, and there were characterestics findings of of Potters’ facies, marked edema, and a structurally normal heart. Histopathology showed changes of renal tubular dysgenesis with the renal cortex containing crowded glomeruli separated by small tubules with distal tubular morphology and absence of proximal tubules (Fig. 1). The proximal tubules should be as numerous as the glomeruli and have plump lining cells with abundant cytoplasm. The medulla appeared largely unremarkable. The family history of parental consanguinity and the severity of symptoms prompted enrolling the family in the Mendelian disease program at Sidra Medicine (Fig. 2a) [13]. Genome sequencing was performed on all family members, and following our in-house analysis pipeline [14], the patient was, initially, found to carry six de novo and nine homozygous rare protein-altering variants, including two that were predicted to lead to loss-of-function (LoF) (Additional file 1). These two include a variant in *OR1J4* (c.221C > G, p.Ser74*), an olfactory receptor gene not known to be associated with Mendelian disease, and a nonsense previously unreported variant (NM_000685.5; c.415C > T; p.Arg139*) was identified in the Angiotensin II Receptor Type 1 (AGTR1) gene (Table 1). Importantly, LoF variants in this gene have been associated with renal tubular dysgenesis (MIM# 267,430) [8]. Both parents were heterozygous carriers of the variant (Fig. 2b) and *in-silico* pathogenicity scores predicted it to be highly damaging (CADD of 39 and GERP of 5.8).

**Table 1:** *AGTR1* reported variants and relevant information

	Present study	Demirgan (2020)	Gribouval(2012)	Gribouval (2012)	Gribouval (2005)
Variant	c.415C > T p.Arg139*	c.376C > T p.Arg126*	c.376C > T p.Arg126*	c.251G > A p.Trp84*	c.110_111insT p.Ile38HisfsX37 c.845C > T p.Thr282Met
dbSNP id	rs1417391173	rs397514687	rs397514687	rs398122935	rs387906577; rs104893677
Variant Effect	Stop gained	Stop gained	Stop gained	Stop gained	Missense
Zygosity	Homozygous	Homozygous	Homozygous	Homozygous	Compound heterozygotes
Exon	3	3	3	3	3
Gender	F	M + F (siblings)	– + M (siblings)	F	M + F (siblings)
Country of origin	GCC	Turkish	Pakistan	North Africa	Europe
Consanguinity	Yes	Yes	Yes	Yes	No
No. Affected	1	2	2	1	2
GA (weeks)	36	M (39) F (32)	– (27) M (37)	37	M (35) F (TP at 24)
Oligohydramnios (weeks)	Yes	M (–) F (20)	Yes	20	M (30) F (24)
Age of death	Day 4	M (Alive) F (Alive)	Both Day 1	Stillborn	M (36 d) F (TP)
Anuria	Yes	M (no) F (Yes, resolved)	Yes	Yes	Yes

“– “ indicate not available

*GA* Gestational age, *TP* Termination of pregnancy

* Termination

## Discussion and conclusion

The molecular mechanisms underlying the genetic basis of RTD pathogenesis are still not fully elucidated; however, LoF/structural variants in genes encoding components of the RAS pathway are a major cause of the disease [8]. Disruption of the RAS leads to defects in the differentiation of proximal tubules during fetal development resulting in severe symptoms during pre- and postnatal periods including fetal anuria and oligohydramnios [15].

In this report, we present a case of a newborn female patient who suffered from congenital RTD and several severe complications, ultimately resulting in perinatal death at five days of life. Genetic analysis of the child and her parents identified a pathogenic nonsense variant in exon 3 of the *AGTR1* gene. The predicted effects of this variant are protein truncation and possibly nonsense-mediated mRNA decay. To date, only eight RTD patients, including ours, have been reported with five different *AGTR1* gene variants (Table 1, Fig. 2c), reflecting the rare nature of RTD and the significance of RAS signaling pathway in early development.

The genetic association of *AGTR1* variants with an RTD phenotype is supported by the literature in which patients suffer severe symptoms during pre-and/or postnatal life [8, 9]. In addition, recent evidence has pointed to the possibility of a milder form of the RTD depending on the variant position in the *AGTR1* gene [16]. A male carrier of a homozygous LoF variant (p.Arg216*) in *AGTR1* has been described who lived to 28 years of age under management with high doses of fludrocortisone which, along with vasopressin, have proved effective in managing RTD [16]. Overall, although the severity of symptoms in patients who carry *AGTR1* mutations is consistent across all reported cases, it has been suggested that, similar to other genetic renal diseases, the phenotype is more severe when the affected protein is located more distally along the RAS pathway [17].

Reaching a final diagnosis of RTD prenatally has been challenging because all prenatal symptoms of oligohydramnios, and IUGR are not specific. This challenge leaves genetic testing as the only viable diagnostic option after none genetic causes have been excluded [18], particularly when offered in the context of prenatal diagnosis through chorionic villus sampling. Even when early symptoms began to emerge postnatally, the patient's instability did not indicate a specific diagnosis. The genetic finding complemented by the histopathology confirmed the diagnosis of RTD. Although the treatment remained supportive, providing prompt answers to healthcare providers and families is immensely valuable.

Given the severity of the condition, improved outcomes for RTD patients can be realized through early detection, facilitating clinical decision making and enhancing neonatal care, particularly in cases of severe congenital diseases with prenatal indications and symptoms. Genetic testing empowers carrier parents to make informed decisions regarding their future family plans. In the case of the newborn discussed here, the parents received appropriate counselling and were informed about the genetic results, and the disease risk in subsequent pregnancies. Early identification of recessive pathogenic variants, particularly in such highly consanguineous population, plays a pivotal role in the success of population screening programs and contributes to lowering the long-term burden of Mendelian diseases.

### Supplementary Information

**Supplementary Material 1.**

## Data Availability

The datasets analysed during the current study are available in the Genome Sequence Archive in Sidra Medicine, Qatar. Variant submitted in ClinVar under accession number VCV002430252.2.

## Abbreviations

ACE: Angiotensin-converting enzyme
AGTR1: Angiotensin II Receptor Type 1
CPAP: Continuous positive airway pressure
CRRT: Renal replacement therapy
ECMO: Extra Corporeal Membrane Oxygenation
IUGR: Intra-uterine growth retardation
PPHN: Persistent pulmonary hypertension of newborn
RAS: Renin angiotensin system
RTD: Renal tubular dysgenesis
