# Supplementary material for: A loss-of-function AGTR1 variant in a critically-ill infant with renal tubular dysgenesis: case presentation and literature review
Source: BMC Nephrol. 2024 Apr 22;25:139. doi: 10.1186/s12882-024-03569-z (PMC11034062; doi:10.1186/s12882-024-03569-z)
Supplement: Supplementary file 1 — Supplementary Material 1. [file 12882_2024_3569_MOESM1_ESM.docx]

**Additional File 1: rare homozygous variants in affected patient.**

| **Chr:position** | **Gene** | **rsid** | **Effect** | **HGVS_C** | **HGVS_P** | **OMIM phenotype** | **MIM number** |
| --- | --- | --- | --- | --- | --- | --- | --- |
| 3: 148459237 | *AGTR1* | rs1417391173 | Stop gained | c.415C>T | p.Arg139* | Renal tubular dysgenesis | 267430 |
| 5: 82837517 | *VCAN* | rs754282657 | missense | c.8695G>A | p.Glu2899Lys | Wagner syndrome 1 | 143200 |
| 5: 156184744 | *SGCD* | NA | missense | c.728A>G | p.Lys243Arg | Muscular dystrophy, limb-girdle, autosomal recessive 6; Cardiomyopathy, dilated, 1L | 601287; 606685 |
| 9: 125281640 | *OR1J4* | rs769943830 | Stop gained | c.221C>G | p.Ser74* | No omim phenotype | NA |
| 9: 131768857 | *NUP188* | rs766451988 | missense | c.5150C>T | p.Ser1717Leu | Sandestig-Stefanova syndrome | 618804 |
| 12: 80665473 | *OTOGL* | rs183159689 | missense | c.2537C>T | p.Pro846Leu | Deafness, autosomal recessive 84B | 614944 |
| 13: 24243133 | *TNFRSF19* | rs369698575 | missense | c.1142C>T | p.Thr381Ile | No omim phenotype | NA |
| 13: 98672038 | *IPO5* | rs149346936 | missense | c.3094A>C | p.Asn1032His | No omim phenotype | NA |
| 14: 20898365 | *KLHL33* | rs1594397551 | missense | c.470C>T | p.Ala157Val | No omim phenotype | NA |

NA: not available

The parents are heterozygous for the above variants.
